# Ectopic Adrenocorticotropic Hormone Syndrome Secondary to Undifferentiated Pleomorphic Sarcoma of the Thigh: A Rare Paraneoplastic Manifestation

**DOI:** 10.7759/cureus.94397

**Published:** 2025-10-12

**Authors:** Gizem Guney, Ibrahim Yildiz

**Affiliations:** 1 Medical Oncology, Acibadem University, Istanbul, TUR

**Keywords:** ectopic acth syndrome (eas), ifosfamide delirium, paraneoplastic syndrome, refractory hypokalemia, undifferentiated pleomorphic sarcoma (ups)

## Abstract

Undifferentiated pleomorphic sarcoma (UPS) is a rare, high-grade soft tissue sarcoma. Ectopic adrenocorticotropic hormone (ACTH) syndrome is an uncommon paraneoplastic phenomenon and is most frequently associated with neuroendocrine tumors. Herein, we present a unique case of a 55-year-old woman diagnosed with UPS of the left thigh, complicated by paraneoplastic ectopic ACTH syndrome. To our knowledge, this is the first reported case of ectopic ACTH syndrome associated with UPS in the adult population.

## Introduction

Sarcomas account for less than 1% of all malignant tumors [1]. Undifferentiated pleomorphic sarcoma (UPS), formerly known as malignant fibrous histiocytoma, is a highly aggressive soft tissue sarcoma [2]. UPS typically arises from mesenchymal stem cells or muscle tissue, and rarely from bone. These tumors often exhibit complex genomic alterations [3]. Paraneoplastic syndromes are complex systemic manifestations from a malignancy, causing an altered immune system [4]. These syndromes may evolve from immunologic or non-immunologic mechanisms. Ectopic adrenocorticotropic hormone (ACTH) syndrome, one of the most common endocrine paraneoplastic syndromes, is defined as Cushing’s syndrome caused by ACTH secretion from non-pituitary tumors, accounting for 5%-20% of all Cushing's cases [5]. The most common sources are lung and mediastinal tumors, followed by gastroenteropancreatic neuroendocrine tumors and pheochromocytoma [6]. Reports of ectopic ACTH syndrome secondary to sarcomas are exceedingly rare, especially in adults. 

## Case presentation

Clinical history and examination

**Figure 1:**
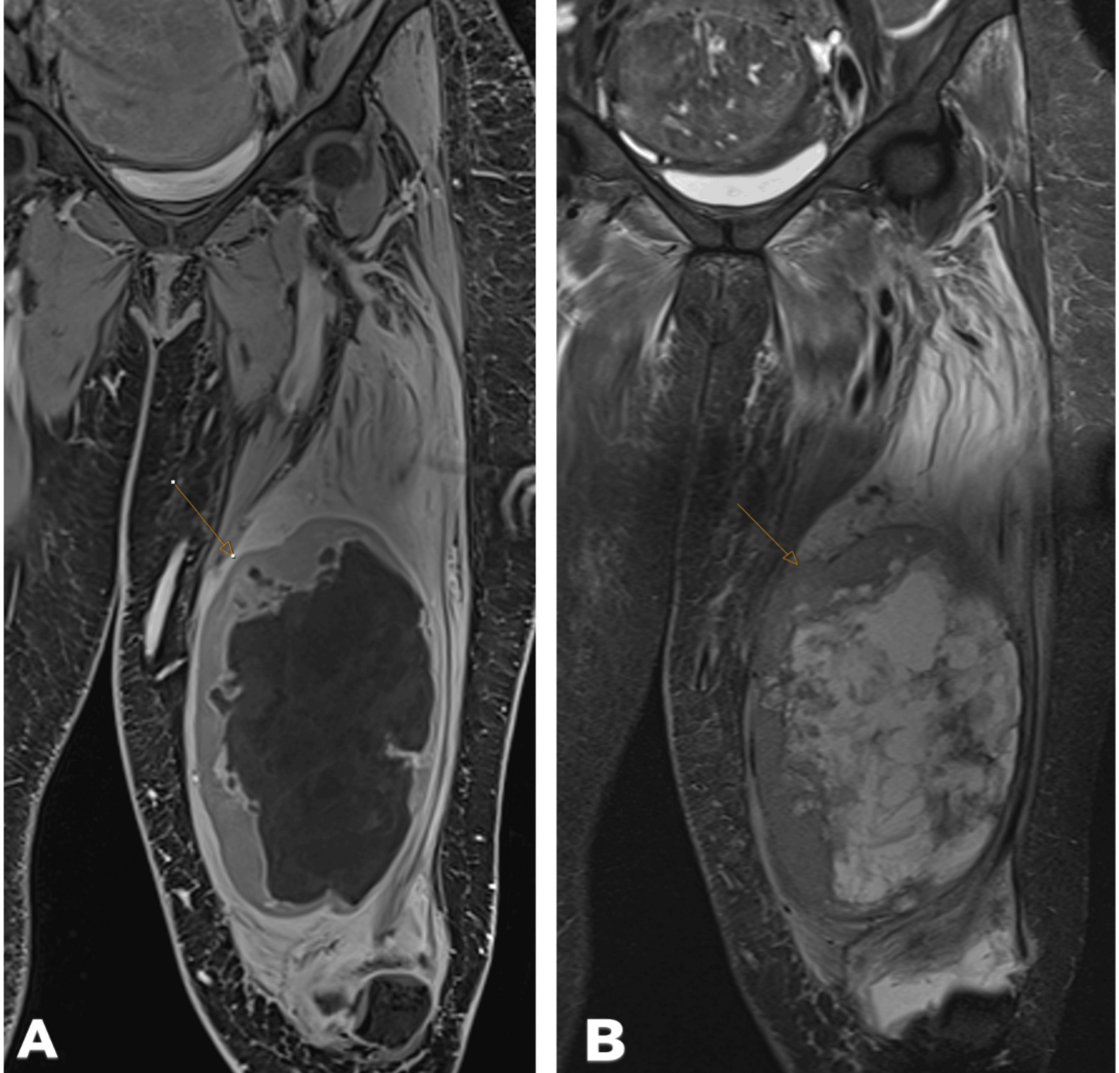
MRI of the mass. A: T2-weighted TSE STIR coronal image. B: Post-contrast T1-weighted VIBE STIR coronal image. The mass measures 225 mm × 78 mm × 74 mm and demonstrates central necrosis (indicated by the arrow) TSE: turbo spin-echo; STIR: short Tau inversion recovery; VIBE: volumetric interpolated breath-hold examination; MRI: magnetic resonance imaging

A 55-year-old woman presented with a rapidly enlarging mass in the left upper anterior thigh. Magnetic resonance imaging (MRI) revealed a 225 mm x 78 mm x 74 mm malignant mass with central necrosis and marked enhancement in the left vastus intermedius muscle (Figure 1). No vascular or lymph node involvement was detected.

Pathological findings

Core needle biopsy demonstrated undifferentiated pleomorphic sarcoma. Immunohistochemistry was negative for cytokeratin AE1/AE3, desmin, myogenin, and S-100, but positive for FLI-1 and H3K27me3 (Table 1).

**Table 1:** Pathology findings from tru-cut biopsy of the patient’s mass UPS: undifferentiated pleomorphic sarcoma

Section	Details
Clinical findings	Left thigh soft tissue mass (20 cm × 10 cm), necrotic, and tru-cut biopsy
Macroscopic findings	10 tissue fragments; sizes ranging from 2.0 cm to 0.6 cm
Immunohistochemical study	Cytokeratin AE1/AE3 (PanCK): negative; desmin: negative; myogenin: negative; H3K27me3: positive (no loss of expression); S-100: negative; and FLI-1: positive (in few scattered cells)
Diagnosis	The specimen lacked distinct morphological features of differentiated sarcoma subtypes. It tested negative for key differentiation markers, including desmin, myogenin (for rhabdomyosarcoma), and FLI1 (for Ewing sarcoma). Due to the absence of clear immunohistochemical differentiation, it was classified as an UPS

Staging and initial management

Staging with fluorodeoxyglucose (FDG) PET-CT and brain MRI showed no evidence of metastasis. The multidisciplinary tumor board recommended neoadjuvant chemoradiotherapy due to the large tumor size, precluding limb-sparing surgery. Adriamycin and ifosfamide chemotherapy was initiated.

Complications and paraneoplastic syndrome

During the first cycle of chemotherapy, the patient developed acute delirium, which was attributed to ifosfamide neurotoxicity and resolved after discontinuation of ifosfamide. She was hospitalized for severe hypertension, tachycardia, profound hypoalbuminemia (1.4 g/dL), hypokalemia (2 mmol/L), hypomagnesemia (1.52 mg/dL), and pancytopenia (Table 2). Despite supportive therapy, she developed generalized edema, bilateral pleural effusions (left: 7 cm, right: 9 cm), and pericardial effusion.

**Table 2:** Laboratory findings of the patient DST: dexamethasone suppression test; ACTH: adrenocorticotropic hormone

Laboratory parameter	Result	Reference range
Albumin	1.4 g/dL	3.5-5.0 g/dL
Potassium	2 mmol/L	3.5-5.0 mmol/L
Magnesium	1.52 mg/dL	1.7-2.2 mg/dL
Morning cortisol (1 mg DST)	35.9 μg/dL	<1.8 μg/dL (after DST)
ACTH	50.8 pg/mL	10-60 pg/mL
Baseline morning cortisol (before 8 mg DST)	14.2 μg/dL	
Morning cortisol (after 8 mg DST)	17.1 μg/dL	Expected: >50% suppression from baseline cortisol

Given the clinical picture, Cushing’s syndrome was suspected. A 1 mg overnight dexamethasone suppression test (DST) revealed a morning cortisol of 35.9 μg/dL. ACTH level was 50.8 pg/mL, consistent with ACTH-dependent Cushing’s syndrome. An 8 mg DST was performed. The morning cortisol level was 14.2 μg/dL before the test, and the morning cortisol level measured after the 8 mg DST was found to be 17.1 μg/dL. The patient's hypercortisolism was attributed to ectopic ACTH syndrome based on these laboratory results. The source was attributed to UPS, as no other lesions were identified.

Neurological status

The patient’s neurological status was notable for the development of acute delirium during the first cycle of chemotherapy, which was attributed to ifosfamide-induced neurotoxicity. The delirium resolved after discontinuation of ifosfamide. No further neurological deficits were observed during follow-up, and there was no evidence of central nervous system metastasis on imaging.

Further management and outcome

Due to persistent poor performance status despite supportive care, Adriamycin and ifosfamide chemotherapy were discontinued. Weekly chemotherapy in combination with radiotherapy was planned.

## Discussion

Sarcomas represent less than 1% of all malignancies [1]. UPS is a high-grade, aggressive soft tissue sarcoma, most commonly arising from mesenchymal or muscle tissue [2]. These tumors are characterized by complex genetic alterations [3].

Ectopic ACTH syndrome is a rare paraneoplastic manifestation, most commonly associated with neuroendocrine tumors, and accounts for 5%-20% of Cushing’s syndrome cases [5,6]. The most frequent sources are lung and mediastinal tumors, with sarcomas being exceedingly rare causes. In the literature, only a few pediatric cases of ectopic ACTH syndrome secondary to Ewing sarcoma and renal clear cell carcinoma have been reported [7-9]. To our knowledge, this is the first reported case of ectopic ACTH syndrome associated with UPS in an adult.

This case highlights the importance of considering paraneoplastic endocrine syndromes in patients with aggressive sarcomas and atypical clinical presentations. Early recognition and management of paraneoplastic syndromes are crucial for optimizing patient outcomes.

## Conclusions

We report a rare case of ectopic ACTH syndrome secondary to UPS of the thigh in an adult woman. This unusual paraneoplastic manifestation broadens the clinical spectrum of tumors capable of producing ectopic ACTH and causing Cushing’s syndrome. The case highlights the importance of maintaining a high index of suspicion for hormonal paraneoplastic syndromes in patients presenting with unexplained metabolic derangements and rapid clinical deterioration, even in the setting of rare malignancies such as UPS.
